# 

**Published:** 1898-06

**Authors:** 


					﻿WTABLI8HtD IS55.
SUBSCRIPTION. $2.00.
ATLANTA
MEDICAL AND SWIGB :
JOURNAL. juL-wtffflf
VOLUME XV
NEW SERIES.
Atlanta, Ga., June, 1898.
No. 4.\f
				

